# Occurrence of bacteraemia following oral and maxillofacial surgical procedures in Port Harcourt, Nigeria

**DOI:** 10.4314/ahs.v21i4.24

**Published:** 2021-12

**Authors:** Omokaro Osaiyuwu

**Affiliations:** Department of Oral and Maxillofacial Surgery, Rivers State University Teaching Hospital, Port Harcourt, Rivers State, Nigeria

**Keywords:** Bacteremia, bacteria isolates, antimicrobial prophylaxis, Port Harcourt, Nigeria

## Abstract

**Study Design:**

A prospective study of 130 patients attending the Government Dental and Maxillofacial Hospital (now Maxillofacial unit, Rivers State University Teaching Hospital), Port Harcourt, between August 2018 and September 2019.

**Objective:**

To examine the factors that affect the occurrence of a bacteremia associated with oral and maxillofacial surgical procedures, and the types of bacteria causing this bacteremia, and establish the need or otherwise for prophylactic antibiotics in, ‘at risk’ patients.

**Methods:**

130 healthy adult patients requiring various oral and maxillofacial surgical procedures under local anesthesia using 2% lidocaine with 1: 100,000 adrenaline, were screened bacteriologically to determine the occurrence of a bacteremia. 5 milliliters of venous blood was collected from the antecubital vein of each patient preoperatively and within 3 minutes postoperatively. The samples collected were cultured and bacteriological identification done and analyzed.

**Results:**

Bacteremia was found in 43 (33.1%) of 130 patients postoperatively. In patients undergoing extractions, bacteremia occurred more frequently when teeth were extracted due to inflammatory dental diseases. There was no statistical correlation between the occurrence of a bacteremia and the number of teeth extracted (p>0.05). Of the 70 isolates obtained postoperatively, 5 (6.4%) were aerobes, 51 (65.4%) were facultative anaerobes (including microaerophiles) and 14 (17.9%) were anaerobes. Among the facultative anaerobes (including microaerophiles), the most frequently isolated bacteria genera were species of *Staphylococcus* (25.7%), *Streptococcus spp*. (17.1%) and *Streptococcus viridans* (15.7%) and among the anaerobes, Bacteriodes spp. (8.6%) were the most frequently isolated. All the isolates were sensitive to azithromycin, amikacin, imipenem and meropenem. However, 3 (30%) of the isolates were resistant to amoxicillin, which is a commonly used drug for antimicrobial prophylaxis.

**Conclusion:**

This study shows the occurrence of bacteremia in Nigerians following various oral and maxillofacial surgical procedures and that the observed bacteremia was not dependent on the patient's age or gender. This study emphasizes the need for antibiotic prophylaxis in those patients who are at risk of developing complications from bacteremia. Amoxicillin as this study shows will not be an effective antibiotic prophylactic agent in a majority of patients. The author therefore recommends the use of azithromycin as an alternative prophylactic agent in those patients.

The presence of bacteria in the blood stream is referred to as bacteremia. In a healthy human, the blood stream is generally considered a sterile environment. Although bacteria nutrients are plentiful in blood, major anti-microbial defenses, which include the circulating neutrophils and monocytes capable of phagocytosis, and the supporting components of humoral immunity including complements and immunoglobulins, help to mop up any bacteria that enter the blood stream.^1^

The oral environment is colonized by a number of species of bacteria, residing therein as commensals. They include anaerobes such as *Peptostreptococcus, Propionibacterium*, *Bacteriodes, Prevotella* and *Fusobacterium* spp., as well as facultative anaerobes such as *Streptococcus, Actinomyces* and *Staphylococcus* spp.^2^,^3^,^4^,^5^. Dental procedures, particularly dental extractions have been documented to cause the introduction of such oral bacteria into the blood stream.^4^,^5^,^6^,^7^

The occurrence of bacteremia following dental procedures has been documented^2^. Cawson, 1991 found that dental extractions regularly released bacteria from the gingival margins into the blood stream and was the first recognized source of bacteremia in dental practice^6^. Extractions are effective in producing bacteremia because of the immense number of bacteria surrounding the teeth and because extraction has a pumping action on the vessels of the periodontal region as they are alternately stretched and compressed by rocking of the tooth in the socket.^6^

The reported occurrence of bacteremia differs from study to study. Rahn et al., 1995, attributed this to factors such as the culture media and methods employed for anaerobic culture, the type of procedures performed, and the time of blood sampling^7^.

The occurrence of bacteremia after oral and maxillofacial procedures does not lead to any complication in healthy individuals. It can however, create serious problems in certain patients^7^. In individuals with congenital or acquired heart disease or those fitted with a valvular prosthesis, circulating bacteria may reach the defective endocardium and cause bacterial endocarditis^8^. Bacteremia can also be fatal in the elderly and children^9^, ^10^, patients on immunosuppressant for organ transplant^11^, patients with uncontrolled diabetes^12^ and those with other valvular heart disease^6^. Bacteremia can also be fatal in patients with prosthetic joint replacement^13^ and in those with other body prostheses such as penile prosthesis^14^.

A mycotic aneurysm of the right carotid artery was reported in a 70-year-old man immediately after a complicated third molar extraction^15^. Ngapeth-Etoundi et al.^16^ reported septicemia and post-extraction coma in three patients. Kroppenstedt et al.^17^ reported a rare finding of an abscess in a pituitary tumor in a 69-year-old man four days after a tooth extraction. Larkin and Scott reported an unusual case of metastatic para spinal abscess and paraplegia as a consequence of simple dental extractions and subsequent bacteremia from a neglected mouth.^18^.

The literature is unclear about the nature and incidence of bacteremia from other oral surgical procedures, and the relationship of such bacteremia to dental disease. This study therefore seeks to establish the occurrence of bacteremia following various oral and maxillofacial surgical procedures, identify the implicated bacteria, and the effect of age, gender and types of dental pathology on such bacteremia. Furthermore, the study will determine the antibiogram of the implicated bacteria. It would also seek to establish the need or otherwise for prophylactic antibiotics in patients who are at risk of developing complications as a result of bacteremia.

## Materials and Methods

This prospective study was conducted at the Government Dental and Maxillofacial Hospital (now Maxillofacial unit, Rivers State University Teaching Hospital), Port Harcourt. Port Harcourt is the capital city of Rivers State in the oil rich region of the Niger delta, southern Nigeria. It is located on latitude 4°46′38.71″ N and longitude 7°00′48.24″ E. The hospital is a tertiary health facility that serves as a referral center for Rivers state and the neighboring states of Bayelsa, Abia and Akwa Ibom.

A total of 130 healthy medically fit adults aged 18–68 years attending the hospital between the periods of August 2018 and September 2019, were sampled for this study. Patients, who have had antibiotics two weeks prior to presentation to the hospital, were excluded. Informed consent was obtained from the patients. Ethical clearance was obtained from the Ethics and research committee of the hospital, and informed consent was obtained from the patients. Patients were financially responsible for their treatment.

SPSS version 21, software was used for statistical analysis. Chi- square test was used to determine the relationship between dependent and independent variables. P value <0.05 was used to indicate significant association. Results are presented in tables 1 to 4.

**Table 1:** Demographic and operative characteristics and the occurrence of Bacteremia

Variable	Frequency	Percent %	Occurrence of Bacteremia n (%)		χ2	P- value
			Yes	No		
**Age (N=130)**						
<25	57	43.9	17 (39.5)	40 (46.0)	0.674	0.714
25–49	54	41.5	20 (46.5)	34 (39.1)		
≥50	19	14.6	6 (14.0)	13 (14.9)		
**Sex (N =130)**						
Male	43	33.1	19 (44.2)	24 (27.6)	3.582	0.058
Female	87	66.9	24 (55.8)	63 (72.4)		
**Procedure** **(N=130)**						
Extraction	44	33.8	15 (34.9)	29 (33.3)	21.181	0.001
Surgical extraction	18	13.8	5 (11.6)	13 (14.9)		
I & D	18	13.8	12 (27.9)	6 (6.9)		
IMF	16	12.3	8 (18.6)	8 (9.2)		
Incisional biopsy	22	16.9	3 (7.0)	19 (21.8)		
Excisional biopsy	12	9.2	0 (0.0)	12 (13.8)		
**Duration of** **procedure** **in mins (N=130)**						
<30	65	50.0	19 (44.2)	46 (52.9)	3.100	0.376
30–59	45	34.6	14 (32.6)	31 (35.6)		
60–90	10	7.7	5 (11.6)	5 (5.7)		
>90	10	7.7	5 (11.6)	5 (5.7)		
**Indications for** **extradiction (N =62)**						
Inflammatory diseases			17 (85.0)	28 (66.7)	2.288	0.130
Non-inflammatory diseases			3 (15.0)	14 (33.3)		
**Number of teeth** **extracted (N=62)**						
1			18 (90)	36 (85.7)	0.221	0.638
>1			2 (10)	6 (14.3)		

Mean Age= 31.3 ± 14.2 years (Range: 15–18)

Mean duration of procedure = 35.9 ± 29.5 mins (Range: 6–150)

**Table 2:** Number and types of bacteria isolates from bacteraemia associated with sex

	Sex		TOTAL
	Male	Female	n (%)
**Aerobes**			
Providencia spp	2	1	3 (4.8)
Alcaligenes	0	2	2 (3.2)
**Microaerophils**			
Staph aureus	7	10	17 (27.4)
Strep viridans	2	7	9 (14.5)
Diphtheroides	1	1	2 (3.2)
Strep spp	6	4	10 (16.1)
Coliforms	4	1	5 (8.1)
**Anaerobes**			
Bacteroides spp	1	5	6 (9.7)
Anaerobic strep	3	1	4 (6.5)
Clostridium spp	1	3	4 (6.5)
TOTAL n (%)	27	35	**62**

**Table 3:** Association between duration of procedure and occurrence of bacteraemia

Variable	Occurrence of bacteraemia		X^2^	P-value
	Yes (n=43)	No (n=87)		
	M±SD	M±SD		
Duration of procedure in patients that had extraction	14.8±6.37	14.9±6.17	-0.049	0.961
Duration of procedure in patients that had surgical extraction	25.4±6.77	29.5±9.33	-0.881	0.391
Duration of procedure in patients that had I & D	38.5±9.56	37.5±5.24	0.237	0.816
Duration of procedure in patients that had IMF	103.1±15.80	105±26.73	-0.171	0.867
Duration of procedure in patients that had biopsy	35.0±22.9	33.7±13.68	0.144	0.886

M = Mean, SD = Standard deviation

**Table 4:** Antibiotic susceptibility pattern of bacterial isolates

Organism isolated in the study	Antibiotics Tested								
	PEN	AMK	AMX	AZT	BAC	CIP	MZT	IMP	MER
**Aerobes**									
Providencia spp	S	S	S	S	S	S	S	S	S
Alcaligenes	S	S	S	S	S	S	R	S	S
**Microaerophils**									
Staph aureus	R	S	R	S	S	I	S	S	S
Strep viridans	S	S	S	S	S	S	S	S	S
Diphtheroides	R	S	S	S	R	S	S	S	S
Strep spp	S	S	S	S	S	S	S	S	S
Coliforms	S	S	S	S	S	S	S	S	S
**Anaerobes**									
Bacteroides spp	R	S	I	S	R	I	S	S	S
Anaerobic strep	R	S	S	S	S	I	S	S	S
Clostridium spp	R	S	R	S	R	R	S	S	S

S=Sensitive, I=Intermediate, R=Resistance

PEN=Penicillin, AMK=Amikacin, AMX=Amoxicillin, AZT=Azithromycin, BAC= Bacitracin, CIP= Ciprofloxacin, MZT= Metronidazole, IMP= Imipenem, MER= Meropenem

### Collection of Samples

Peripheral blood samples were obtained from each patient by venepuncture of the antecubital vein of the left arm. 5 milliliters of venous blood was obtained aseptically preoperatively and another 5 milliliters within 3 minutes of completion of the procedure, and transferred into separate blood culture bottles containing nutrient broth and thioglycollate broth. Nutrient broth cultures were incubated aerobically, while the thioglycollate broth cultures were incubated anaerobically in gas pak jars at 37°C for 10 days.

Cultures with evidence of growth as indicated by turbidity and, or hemolysis were sub cultured. The nutrient broth cultures were sub cultured unto plates of blood agar, chocolate agar and MacConkey agar. The blood and MacConkey agar plates were incubated aerobically while the chocolate agar plates were incubated under increased carbon dioxide tension in candle jars at 37 degrees Celsius for 48hrs. Thioglycollate blood cultures were sub cultured onto plates of blood agar and incubated anaerobically in gas pak jars for 48 hours. Bacterial isolates were identified by colony morphology, gram staining reaction, biochemical tests using catalase test, coagulase test, citrate utilization test (TSI) (OXOID, UK), Urease test (BBL™) and motility indole lysine (MIL)(BBL™) tests as outline by Cowan and Steel, 1993^19^.

### Antimicrobial sensitivity testing

The Kirby- Bauer disk diffusion method was used to test bacteria susceptibility to antimicrobial agents. Commercially available multidisks (Sigma chemicals) containing the following antimicrobials: penicillin (10µg), amikacin (10µg), ciprofloxacin (5µg), azithromycin (15µg), bacitracin (10µg), amoxicillin (20µg), metronidazole (30µg), imipenem (10µg), meropenem (20µg), were used on diagnostic sensitivity agar plate cultures of each isolate. Cultures were incubated as described earlier, and zones of inhibition after incubation were interpreted as outlined by Bauer et al., 1966^20^.

## Results

A total 130 patients (mean age: 31.3± 14.2 years; range: 15–68 years) attending the Government dental and Maxillofacial Hospital (now maxillofacial unit of the Rivers State University Teaching Hospital), Port Harcourt were studied.

There were 43 (33.1%) males (mean age: 34.2± 16.6, range 18–68) and 87 (66.9%) females (mean age: 29.9± 12.8, range 15–65). Forty- four (33.8%) patients had intra-alveolar extractions, 18 (13.8%) patients had surgical extraction, 22 (16.9%) patients had incisional biopsy, 12 (9.2%) patients had excisional biopsy, 16 (12.3%) patients had inter-maxillary fixation done and 18 (13.8%) patients had incision and drainage.

Five (3.8%) cultures of blood samples taken preoperatively were positive. However, after surgery, bacteremia was found in 43 (33.1%) of the 130 patients sampled. Bacteremia was found in 19 (44.2%) of 43 males, and in 24 (55.8%) of 87 females. There was no statistical significant difference in the occurrence of bacteremia between males and females p=0.058.

A total of 70 isolates were obtained from the survey postoperatively. 5(6.4%) of the isolates were aerobes, 51(65.4%) were facultative anaerobes or microaerophiles and 14 (17.9%) were anaerobes. The most frequently isolated anaerobes were Bacteriodes (6), followed by anaerobic Streptococcus spp. (4) and Clostridium spp. (5). Staphylococcus aureus (18) and non- hemolytic Streptococcus spp. (12) were the most frequently isolated, followed by α- hemolytic *Streptococcus* spp. (11) among the facultative anaerobes.

27 (43.5%) bacteria were isolated in males and 35 (56.5%) were isolated in females. More bacteria were isolated in females than males though the difference was not statistically significant (p=0.058 χ^2^ =3.58). The occurrence of bacteremia was higher in those that had extraction as a result of inflammatory dental diseases 17 (85%) than in those that had extractions as a result of non- inflammatory diseases 3 (15%). This difference was however not statistically significant (p=0.130 χ^2^ =2.29).

The mean duration of procedure was 35.9± 29.5 (range 6–150). P=0.376 χ^2^ =3.10

## Discussion

The result of this study shows a 3.8% bacteria isolation rate preoperatively, which agrees with the observation of Guntheroth^22^ and that of Hogevik^23^. This could have been caused by the normal activities of gum chewing, brushing of teeth and eating carried out by the patients preoperatively, leading to the production of micro movements of the teeth within the sockets, thus, provoking bacteremia. In addition to the organisms that were isolated pre operatively from these patients, other genera were cultured postoperatively. This indicates that the surgical procedures provoked the introduction of additional bacteria into the blood stream.

Bacteria isolation rate of 33.1% was found postoperatively in patients studied. This is comparable with the result of the study by Osaiyuwu et al.,^5^. Comparable bacteria isolation rates were obtained in two, separate studies by Roberts et al., who reported an occurrence of 38% and 38.7% following dental extractions in adults and children respectively.^24^, ^25^

In this study, the presence of bacteremia was not age and gender dependent. This observation agrees with the findings of Osaiyuwu et al.^5^ in Nigeria and with that of Robert et al.10 in the United Kingdom. It however contrasts the findings of Okabe et al.,^3^ in Japan and Lockhart et al.,^41^ in the United Kingdom.

In this study the occurrence of bacteremia was found to be dependent on the procedure. There was a significant association between the occurrence of bacteremia and the different oral surgical procedures (p=0.001 χ^2^ 21.18). This finding agrees with those of Osaiyuwu et al.^5^, Robinson et al.^26^ and Okabe et al.^3^. They had identified the degree of surgical trauma, tissue injury and the type of disease the patient presented with as factors that could be responsible for increased occurrence of a bacteremia in patients undergoing extractions. The bacteria isolation rate in this study was particularly high in patients who had extractions and in those who had undergone incision and drainage and intermaxillary fixation. The most traumas to tissue in this study were caused by intermaxillary fixation, incisional biopsies and incision and drainage. Though the combined effect of soft tissue trauma with bone manipulation made inter-maxillary fixation the most traumatic procedure compared to other procedures, they produced less bacteremia than the latter. This was probably so because during incision and drainage soft tissue manipulation led to the introduction of bacteria into the blood stream from an already infected site or focus. This observation shows that bacteremia associated with oral and maxillofacial surgical procedures were not only affected by the degree of surgical trauma but also by the presence of infection at the site of operation. This also explains why patients requiring extractions who presented with inflammatory diseases such as pericoronitis, acute periapical periodontitis and chronic periodontitis had more bacteremia positive cases than those presenting with non-inflammatory diseases such as impacted teeth and supernumeraries.

In this study there was no statistically significant association between the duration of the procedures and the occurrence of bacteremia. This contrasts the findings of Okabe et al.^3^. They found a significantly higher occurrence of a bacteremia when the duration of the procedure lasted more than 100 minutes, than in shorter durations of operation. This is perhaps due to the fact that those procedures that took longer to complete, and that theoretically would have caused more tissue damage, and therefore, more bacteremia were less likely to be infected with the consequent effect of fewer bacteria introduced into the blood stream.

Okell and Elliot first investigated the type of bacteria involved in bacteremia associated with tooth extraction. In those days Streptococcus spp. were the isolates most commonly found in association with tooth extraction^33^. Similar bacteria genera were isolated in this study, facultative anaerobes being the most frequently isolated bacteria. 17.7% of the isolates were anaerobes. This contrasts the study by Otten et al., and those of Lee and Kim, that found anaerobic bacteremia as high as 80% following dental extractions^2^,^34^. Similarly, Baltch et al., while studying bacteremia following dental cleaning in patients, found an anaerobic bacteremia of 74.6% and 25.4% aerobes^35^. The low level of anaerobic bacteremia in this study is similar to the values reported by Roberts et al.,^25^, who investigated bacteremia in children undergoing four procedures used for conservative dentistry and found that 50% of the organisms isolated were facultative anaerobes (Viridans streptococcus). This low level of anaerobic bacteremia is further supported by the work of Amuradha and co- workers^36^, who reviewed cases of anaerobic bacteremia and found that of the 93 blood cultures they received with suspicion of anaerobic bacteremia over a period of two years, only 18.3% showed anaerobic growth.

The low level of anaerobic bacteremia seen is most likely because anaerobes are very fastidious organisms and most of them do not grow well in blood. Culture techniques also determine the level of anaerobic yield with newer and more sophisticated techniques giving higher yield. In this study traditional culture techniques were used and this could partly have accounted for the low yield of anaerobes. This agrees with the findings of Saito et al.,^37^. Of the facultative anaerobes (including microaerophiles) the most frequently isolated genera were *Staphylococcus aureus, Streptococcus* spp., *Streptococcus viridans*, *Diphtheroids* and *Coliforms*. Among the anaerobes, *Bacteroides, anaerobic Streptococcus* and *Clostridium* spp. were more frequently isolated. These isolate are similar to the organisms isolated by Lucas and Roberts.^38^. *Streptococcus* and *Staphylococcus* were the most frequently isolated genera among facultative anaerobes. *Bacteroides* spp. was the most frequently isolated among obligate anaerobes, which agrees with the findings of Otten et al.^2^, Lee and Kim^34^, Okabe et al.^3^, Roberts et al.^25^, Amuradha et al.^36^, Bhatawadekar and Bhardwaj^28^, Saito et al.^37^ and Rajasuo et al.^4^. These organisms are characteristic of odontogenic bacteremia.

The endocarditis working party (EWP) of the British society for antimicrobial chemotherapy recommended that patient with congenital or acquired heart disease undergoing extraction of teeth, mucoperiosteal surgery or scaling should be given systemic antibiotic prophylaxis.^39^,^40^. The guidelines recommend the use of amoxicillin, and in patients allergic to penicillins, clindamycin should be used. In countries where oral suspension of clindamycin is not available, for example, the United Kingdom, azithromycin or clarithromycin can be used.

In this study the antibiotic sensitivity pattern of the isolated bacteria was examined. Azithromycin was found to be effective against both gram positive and gram negative isolates. Osaiyuwu et al.^5^ and Dagnew et al.^42^, reported ciprofloxacin as an effective antimicrobil agent The isolates in this study were found to be resistant to amoxicillin clavulanic acid, which contrasts the findings by Wasihun et al.,^43^ but agrees with the findings of Osaiyuwu et al.^5^. The increased resistant antibiogram pattern of the isolates suggests a high resistance gene pool perhaps due to gross abuse and misuse of antibacterial agents. This is so because these antibacterial agents are readily available in this country as over the counter drugs.

This study is from a single tertiary health facility, and our findings may not have been representative of Port Harcourt city, since the city has another tertiary referral center. However, this study provides an insight into the occurrence of a bacteremia following oral surgical procedures, and the factors that affect such occurrence. One of the limitations of this study was the unavailability of newer blood culture methods such as the Bactec radiometric (460) system and the Bactec 760 system (Becton Dickson UK Ltd Oxford OX43LX United Kingdom). These systems would have ensured a higher culture yield, especially for the anaerobes. These systems detect the carbon dioxide (CO_2_) produced by microorganisms growing in the defined culture medium. The carbon dioxide is measured using a radiometric method in the Bactec radiometric (460) and by infrared spectrophotometry in the Bactec 760 system. In addition, the Streptococci could not be speciated due to lack of the API Strep 20 system. The intensity of the bacteremia could not be determined because of the unavailability of the isolator system, which detects the colony forming units per milliliters of blood.

## Conclusion

This study shows that bacteremia is frequently associated with maxillofacial surgical procedures. The occurrence of such bacteremia can have a deadly effect in those patients who are considered to be at risk of acquiring such infections. Such individuals include those with congenital heart disease, those fitted with a valvular prosthesis, those with defective endocardium and those on immunosuppressant for organ transplant. Therefore, antibiotic prophylaxis should be given to any patient who is suspected to be at risk of developing complications from bacteremia. The observed occurrence of a bacteremia is not affected by the patient's gender or age and that the differences in the occurrence of a bacteremia from procedure to procedure, is probably due to differences in the disease condition necessitating the procedure. The most frequently isolated bacteria were the facultative anaerobes (including microaerophiles). The bacteria isolated are typical of those seen in other reported cases of odontogenic bacteremia. The anaerobic yield was low and finally, the efficacy of amoxicillin as a prophylactic antibiotic is seriously in doubt because of the observed resistance by some bacteria isolates.

The author therefore recommends the use of Azithromycin as the prophylactic antimicrobial of choice in patients who are susceptible to the effects of bacteremia.
